# Impact of gamma irradiation on lemongrass (*Cymbopogon citratus*): phytochemical content, antioxidant and antibacterial activity

**DOI:** 10.1186/s12896-025-01090-1

**Published:** 2026-01-09

**Authors:** Wael El-Desouky Ibrahim, Nermien Z. Ahmed

**Affiliations:** 1 Radiation Protection and Safety Department, Hot Laboratories and Waste Management Centre, Egyptian Atomic Energy Authority, Cairo, Egypt; 2 Molecular Drug Evaluation Department, Egyptian Drug Authority (EDA), Giza, Egypt

**Keywords:** Antibacterial activity, Antioxidant capacity, *Cymbopogon citratus*, Gamma irradiation, Phytochemicals

## Abstract

**Background:**

Gamma (γ) irradiation is a safe, non-chemical treatment used to decontaminate and enhance the biochemical quality of aromatic and medicinal plants. *Cymbopogon citratus* (lemongrass) essential oil (CCEO) is dominated by oxygenated monoterpenes such as citral and geraniol, compounds that confer notable antioxidant and antimicrobial activities. This study’s objective was to assess the effects of gamma irradiation on the phytochemical composition, antioxidant activity, and antibacterial efficacy of CCEO.

**Methods:**

Fresh lemongrass leaves were hydrodistilled to obtain essential oil (EO) then exposed to γ-irradiation at 0, 5, 10, 15, and 20 kGy. GC‒MS analysis was conducted to assess the essential oil composition. Total phenolic compounds (TPC) and total flavonoid content (TFC) were quantified using Folin–Ciocalteu and AlCl_3_ colorimetric assays, respectively. Antioxidant activity was assessed by DPPH^•^ radical scavenging. Antibacterial activity was evaluated against *Staphylococcus aureus* and *Pseudomonas aeruginosa* via the well-diffusion method. All analyses were performed in triplicate.

**Results:**

GC–MS analysis identified 22 constituents dominated by citral isomers. Citral A (geranial) increased from 38.72 to 40.06 %, citral B (neral) from 31.85 to 33.03 %, α-myrcene from 7.08 to 8.05 %, and geraniol from 4.40 to 5.02 % as irradiation increased to 20 kGy. TPC rose from 92.12 to 103.11 mg GAE/g oil (+11.9 %), and TFC from 109.25 to 208.47 mg QUE/g oil (+90.7 %). Antioxidant capacity improved, with IC₅₀ decreasing from 611.28 to 498.08 µg/mL and DPPH scavenging increased from 70.66 % to 75.15 % (at 1000 µg/mL). Antibacterial efficacy also strengthened: inhibition zones expanded from 10.2 mm to 21.4 mm (+110 %) for *S. aureus* and from 8.1 mm to 18.9 mm (+134 %) for *P. aeruginosa*. Moderate irradiation doses (10–20 kGy) produced the greatest enrichment in oxygenated monoterpenes, phenolics, flavonoids, and bioactivity, while avoiding degradation of thermolabile compounds.

**Conclusions:**

Controlled γ-irradiation doses markedly enhanced the compositional and functional quality of CCEO by increasing citral and geraniol content, elevating phenolic and flavonoid contents, and improving antioxidant and antibacterial performance. Optimized irradiation up to 20 kGy represents a practical post-harvest strategy for augmenting the industrial and pharmacological value of CCEO.

**Graphical Abstract:**

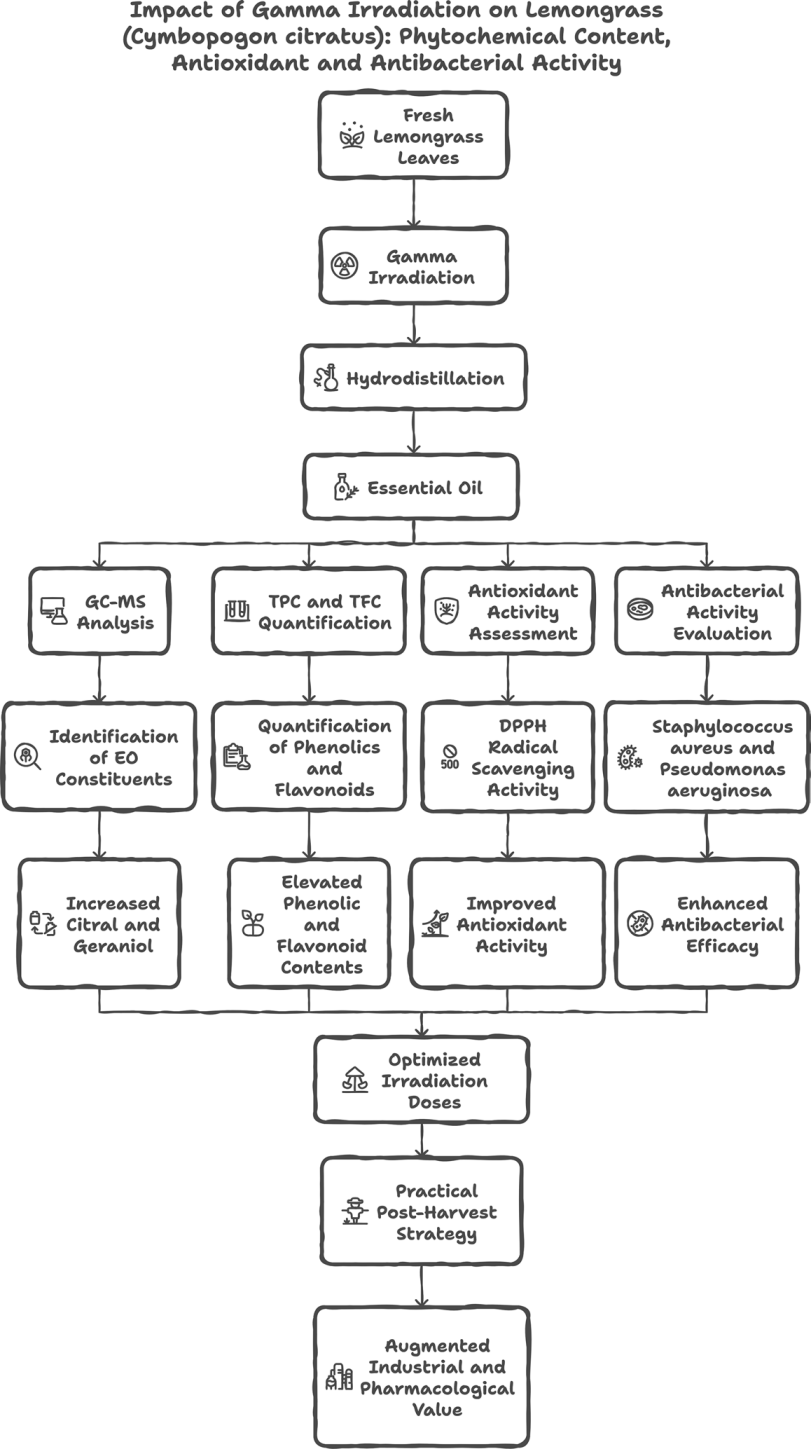

## Highlights

γ-Irradiation at 5, 10, 15, and 20 kGy modified *Cymbopogon citratus* essential oil, increasing citral, geraniol, and β-myrcene levels.

Moderate doses (10-15 kGy) enhanced total phenolic (TPC) and flavonoid (TFC) contents.

Irradiated essential oils showed higher antioxidant activity (lower IC_50_ and stronger DPPH scavenging).

Improved antibacterial efficacy against *Pseudomonas aeruginosa* and *Staphylococcus aureus*.

Controlled γ-irradiation is a safe, green method to enhance the phytochemical quality and functional value of lemongrass essential oil.

## Introduction

*Cymbopogon citratus* (DC.) Stapf, commonly known as lemongrass, is a perennial aromatic grass cultivated widely across tropical and subtropical regions for its essential oil (EO) and applications in food, pharmaceutical, and cosmetic industries [1]. Lemongrass EO is rich in oxygenated monoterpenes, primarily citral, a mixture of the geometric isomers neral and geranial, which impart the characteristic lemon aroma and possess potent antioxidant, anti-inflammatory, antimicrobial, and anticancer effects [2]. Other constituents such as β-myrcene, geraniol, limonene, and linalool contribute synergistically to the biological activity and industrial value of CCEO [3, 4]. The chemical composition and yield of CCEO are influenced by genetic factors, agro-climatic conditions, harvest time, and post-harvest handling [5]. Owing to its versatility and high market demand, CCEO is a prime candidate for sustainable technologies that enhance its quality and stability.

Among these technologies, gamma (γ) irradiation has emerged as a non-thermal, residue free method to improve microbial safety and modulate the phytochemical content of aromatic and medicinal plants [6]. Gamma rays are highly penetrating photons that interact mainly with intracellular water, producing reactive oxygen species (ROS) such as hydroxyl radicals (^•^OH), hydrogen peroxide (H_2_O_2_), and superoxide anions (O_2_^•-^) [7]. At controlled doses, these ROS serve as secondary messengers, triggering oxidative signaling cascades that activate enzymes like phenylalanine ammonia-lyase (PAL), chalcone synthase (CHS), and terpene synthase (TPS) involved in phenolic, flavonoid, and terpenoid biosynthesis [8–10]. Activation of these pathways enhances the antioxidant and antimicrobial potential of plant products.

Recent researches confirm that low-to-moderate doses of γ-irradiation can significantly improve EO yield and biological activity in aromatic herbs. For example, irradiation up to 10 kGy increased thymol and carvacrol in *Thymus vulgaris* [11]. In Moroccan *Tanacetum annuum* EO, exposure to 5–10 kGy altered monoterpene composition, enhanced antioxidant potential, volatile composition and antibacterial activity [12]. Similarly, *Curcuma longa* and *Curcuma alismatifolia* exhibited elevated phenolic levels and improved DPPH radical-scavenging activity following γ-irradiation up to 15 Gy [13]. A recent study on *Satureja mutica* found that 2.5 kGy produced the highest EO yield and phenolic content, while higher doses (7.5–10 kGy) caused declines, illustrating a dose-dependent biphasic response [14]. However, higher doses (above 20 kGy) often cause degradation of thermolabile compounds, lipid oxidation, and reduced biological efficacy, emphasizing the importance of dose optimization [15]. In the *Cymbopogon* genus, irradiation has likewise been linked to improved physiological and biochemical performance. Citronella grass (*Cymbopogon winterianus*) subjected to low γ-doses exhibited enhanced photosynthetic efficiency, antioxidant-enzyme activity, and EO yield [16]. This biphasic or hermetic response reflects a balance between beneficial stress signaling and oxidative injury. Enhanced phenylpropanoid metabolism under irradiation correlates with elevated PAL and peroxidase activity, promoting accumulation of phenolics and flavonoids that scavenge radicals and stabilize essential oils [6, 7]. Moreover, irradiation can improve antimicrobial activity by elevating aldehydes and alcohols such as citral and geraniol the key antimicrobial molecules in CCEO [2, 4].

Despite these promising reports, the mechanistic response of lemongrass EO to γ-irradiation remains poorly characterized. Previous studies primarily focused on microbial decontamination or yield optimization, with limited insight into the biochemical and compositional changes induced by γ-irradiation. Consequently, there is a clear knowledge gap regarding how incremental γ-irradiation doses affect lemongrass essential oil yield and terpene distribution (monoterpenes vs. oxygenated monoterpenes), and how such changes translate into measurable antioxidant (DPPH) and antibacterial outcomes. To address this, the present study aims to evaluate the effects of graded γ-irradiation doses (5, 10, 15, and 20 kGy) on the phytochemical composition, antioxidant capacity, and antibacterial activity of *Cymbopogon citratus* essential oil. This integrated approach provides new insight into the radiochemical stability and functional enhancement of *C. citratus* essential oil, offering practical implications for its biotechnological and industrial applications.

## Materials and methods

### Sources of plants and bacteria

Fresh LG plants were collected from six-month-old plants that cultivated under open-field conditions (temperatures ranging between 22 and 36^°^C) at the experimental farm of the Applied Research Centre of Medicinal Plants (ARCMP), Egyptian Drug Authority, Egypt. The plant was planted in March 2024 and harvested in September 2024 at the full vegetative stage. Plants were grown in sandy-loam soil (pH 7.2 ± 0.3; organic matter 1.4%) under drip irrigation. Irrigation frequency was every three days, maintaining field capacity at approximately 70%. All plants originated from vegetative clonal propagation of a single mother stock to ensure genetic uniformity. Only healthy and morphologically uniform plants were selected to ensure homogeneity before subsequent extraction and γ-irradiation treatments. Gram-positive (*Staphylococcus aureus*, ATCC 25923) and gram-negative (*Pseudomonas aeruginosa*, ATCC 27853) stock cultures of bacteria were acquired from Al-Azhar University’s Faculty of Science, Regional Center for Mycology and Biotechnology, Cairo, Egypt.

### Chemicals

Folin-Ciocalteu reagent, 2,2-diphenyl-1-picrylhydrazyl (DPPH), gallic acid, sodium carbonate (Na_2_CO_3_), quercetin, aluminum chloride (AlCl_3_), ethanol (absolute), sodium nitrite (NaNO_2_), anhydrous sodium hydroxide (NaOH), dimethyl sulfoxide (DMSO), gentamycin, Nystatin were purchased from Sigma Aldrich Chime, Germany. The Mueller Hinton broth (Difco) and nutrient agar (St. Louis, MO) were obtained from Sigma Aldrich. The GC-MS chemicals were obtained from Sigma Aldrich. Additional analytical-grade reagents (purity > 95%) were used in the tests.

### Essential oil extraction

Fresh aerial parts of *Cymbopogon citratus* were washed with distilled water, blotted dry, and chopped to 1–2 cm segments. For each extraction batch, 500 g of fresh material were loaded into a 2 L round-bottom distillation flask with 5.0 L of distilled water (plant: water ratio 1:10 w/v). Hydrodistillation was performed for 3 h at 100^°^C using a standard Clevenger-type apparatus (vertical condenser; graduated oil trap 0–2 mL with 0.1 mL divisions; side-arm return), with heating set to maintain gentle reflux (boiling rate ca. 4–6 drops/sec at the condenser). Inlet cooling water was maintained to ensure efficient condensation of volatiles. The collected essential oil layer was withdrawn periodically from the graduated trap, dried over anhydrous sodium sulfate (Na_2_SO_4_) (0.5 g per mL oil, vortexed and allowed to stand 30 min). Oils were transferred into glass vials, sealed, and stored at 20^°^C, protected from light, until analysis. Each extraction was carried out in triplicate independent batches using identical settings to verify repeatability [17]. 0.1 mL of essential oil was mixed with 0.9 mL of 90% ethanol, vortexed for 5 min, and centrifuged at 4000 rpm for 10 min. The resulting ethanolic supernatant served as the sample for subsequent assays.

### Irradiation treatment

Fresh harvested LG leaves during October 2024 were exposed to γ-irradiation cumulative doses of 5, 10, 15, and 20 kGy at a dose rate of 1.45 kGy/h with corresponding exposure times of approximately 3.4, 6.9, 10.3, and 13.8 h, respectively using a ^137^Cs gamma chamber 4000-A India irradiation facility at the National Center for Radiation Research and Technology (NCRRT), Egyptian Atomic Energy Authority, Cairo, Egypt.

### Gas chromatography‒mass spectrometry (GC‒MS) analysis of CCEO

A Thermo mass spectrometer detector in conjunction with trace GC ultra-gas chromatographs (THERMO Scientific Corp., USA) (ISQ single quadrupole mass spectrometer) was used. A TG‒WAX MS column (30 m × 0.25 mm i.d., 0.25 μm film thickness) was part of the GC‒MS system. The subsequent temperature program was used to perform the analyses: helium as the carrier gas at a flow rate of 1.0 mL/min and a split ratio of 1:10:4.0^°^C/min to 160^°^C and held for 6 min; rising at 6^°^C/min to 210^°^C and held for 1 min; and 40^°^C for 1 min. A temperature of 210^°^C was maintained for both the injector and detector. Injections of 1 µL of the mixtures were always made using diluted samples (1:10 hexane, v/v). Using a spectral range of m/z 40–450, mass spectra were acquired by electron ionization (EI) at 70 eV. The two main analytical techniques used to identify the constitutions were (a) mass spectra (authentic chemicals, Wiley spectral library collection, and NIST library) and (b) KI and Kovats indices in relation to n-alkanes (C9-C22) matching publicly available data from the National Institute of Standards and Technology [18].

### Total phenolic content

The total phenolic content of irradiated and non-irradiated CCEO was determined using the Folin–Ciocalteu colorimetric method described by Singleton et al. (1999) [19]. Briefly, aliquot of ethanolic CCEO solution (1.0 mg/mL) was mixed with Folin–Ciocalteu reagent (1:10 v/v) and incubated for 5 min in the dark, followed by the addition of 7.5% (w/v) Na_2_CO_3_ to reach a final volume of 3 mL. After 30 min at 25^°^C, absorbance was measured at 725 nm using UV-Visible spectrophotometer against a reagent blank. The outcomes are represented as milligrams of gallic acid equivalent per gram of oil.

### Total flavonoid contents

The total flavonoid content of irradiated and non-irradiated CCEO was determined using a colorimetric assay with aluminum chloride, as detailed in Marinova et al., 2005 [20]. 0.5 mL of CCEO was mixed with 0.3 mL of 5% sodium nitrite. Five minutes later, 0.3 mL of 10% aluminum chloride was added, 2 mL of 1.0 M sodium hydroxide was added after 6 min, and the complete volume with distilled water was made up to 5.0 mL. The absorption of the blend against the reagent blank was recorded at 510 nm using a UV-Visible spectrophotometer (Jasco V-530, Japan). Quercetin was used as a standard; the mean of three readings was used to calculate the total flavonoids content, which was expressed as mg of quercetin equivalent/gram of oil.

### Antioxidant activity of CCEO by scavenging activity by DPPH assay

The antioxidant activity of the CCEO was evaluated via the 2,2-diphenyl-1 picrylhydrazyl (DPPH) radical scavenging activity as outlined by Gulluce et al. (2004) [21]. A stock solution of CCEO was prepared in ethanol (1 mg/mL), and serial dilutions were made to obtain final concentrations of 20, 40, 60, 80, and 100 µg/mL. The DPPH solution (0.1 mM) was prepared via dissolving 3.9 mg DPPH in 100 mL ethanol and used as the blank sample. The solution was kept for 30 min in the dark in order to reaction to be completed. Oil extract (50 µL devolved in 900 µL ethanol) was added into 2 mL of DPPH solution and was incubated in the dark. After 30 min, the absorbance of each solution was determined at 517 nm using a UV-Visible spectrophotometer (Jasco V530, Japan). Ethanol solvent was used as blank. All measurements were performed triplicate to establish their correctness. Ascorbic acid was used as a positive control. The following formula was used to calculate the radical scavenging activity;

$$\begin{aligned}&\mathrm{DPPH}\:\mathrm{scavenging}\:\mathrm{activity}\left(\%\right)\cr&\quad=\frac{\mathrm{A}_{\mathrm{blank}}-\mathrm{A}_{\mathrm{sample}}}{\mathrm{A}_{\mathrm{blank}}}\times100\end{aligned}$$

Where,

A _blank_ = absorption by the blank sample.

A _sample_ = absorption by the samples.

A dose–response curve was constructed by plotting inhibition percentage against concentration, and the IC_50_ value (µg/mL), the concentration required to scavenge 50% of DPPH radicals, was determined by nonlinear regression analysis (GraphPad Prism v9.0). All measurements were performed in triplicate.

### Bacterial culture preparation

The stock bacterial cultures were subcultured into newly made nutrient agar and incubated for 24 h at 37^°^C to create fresh bacterial cultures. To achieve the required cell density of 1.5 × 10^8^ (cells/mL), these microbial cultures were moved into freshly made nutritional broth and standardized via the 0.5 McFarland turbidity standards method via a spectrophotometer (SCO TECH SPUV-19-Germany). All biosafety instructions/precautions were implemented and strictly followed throughout the experimental procedures. After the experiment is over, all bacterial cultures, isolates and remains of potential biological hazards were safely disposed by autoclaving.

### Antibacterial activity assay

Making use of the agar well diffusion technique outlined by [22], the antibacterial activity of both non-irradiated and irradiated CCEO against *S. aureus* and *P. aeruginosa* was evaluated. In summary, nutrient agar plates were covered with 100 µL of bacterial inoculum that contained 1.5 × 10^8^ CFU/mL. A sterile cork borer was then used to create the wells in the media, and 100 µL (10 µg) of the extracts was added to each well. The solvent (10% DMSO) loaded well served as the negative control, while gentamycin and nystatin standard reference antimicrobial discs served as the positive controls. Following a 24-hour incubation period at 37^°^C, the inhibition zones on the bacterially injected plates were measured in millimeters.

### Statistical analysis

Outcomes were revealed as averages ± standard errors, and the analyses were executed in triplicate. One-way analysis of variance and Duncan’s test are two techniques for comparing means (ANOVA). Statistical software version 25 (IBM, Armonk, NY, USA) of the SPSS/PC program was utilized at *p* < 0.05 [23].

## Results and discussion

### Chemical composition of CCEO

The importance of medicinal plants’ and their products’ efficacy, safety, and quality is a key component of contemporary methods for producing and using aromatic and medicinal plants [24]. Exposure to gamma radiation will either increase or decrease the extracted yield of EO and its compounds [25].

**Fig. 1:**
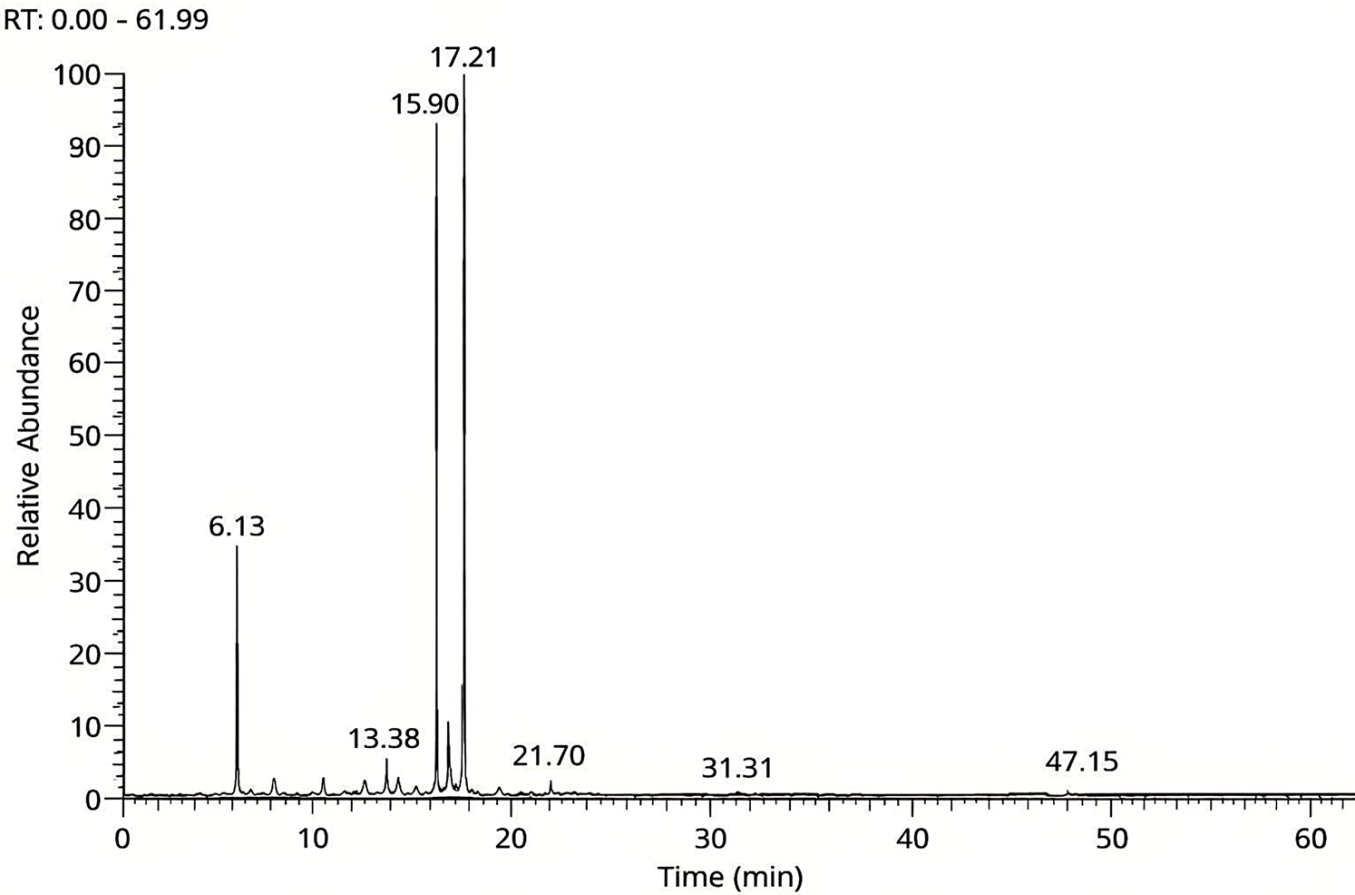
Analysis of lemongrass (*Cymbopogon citratus*) EOs extract by GC-MS

The GC‒MS analysis of CCEO, which is displayed in Fig. 1, revealed 22 components, or 99.96% of the overall composition. Monoterpenes made up 96.37% of the EO, followed by sesquiterpenes at 1.25% and diterpenes at 0.21%. Citral made up the majority of the essential oil (70.57%), with 38.72% geranial and 31.85% neral, followed by myrcene at 7.08%. Additionally, geraniol (4.40%), cis-verbenol (3.15%), cis-ocemene (2.53%), and Bornel (1.60%) were found in the essential oil. Following gamma irradiation, the chemical constituents of CCEO was examined via GC-MS at the following doses: 0 (control), 5, 10, 15, and 20 kGy. Twenty-two volatile compounds, including monoterpenes, sesquiterpenes, and diterpene were identified in all the groups with concentrations given as relative percentages of the total peak area (Table 1).

The CCEO was predominantly composed of citral A (Geranial), citral B (Neral), and α-Myrcene, which together accounted for 70.57% of the total composition across all the treatments. Citral A (Geranial) progressively increased with increasing irradiation dose, rising from 38.72% at 0 kGy to 40.06% at 15 kGy and 20 kGy. Citral B (Neral) followed a similar pattern, increasing from 31.85% (control) to 33.03% at the highest dose. Additionally, the α-Myrcene concentration exhibited a gradual enhancement from 7.08% to 8.05%, suggesting a stimulatory effect of irradiation on these key aldehydes and terpenes. These findings suggest that moderate to high doses of GI may enhance the formation or stability of major oxygenated terpenes, which are primarily responsible for the properties citrus aroma and reported bioactivities of the essential oil.

**Table 1:** GC‒MS analysis of the bioactive constituents of non-irradiated and γ-irradiated CCEO. Data are expressed as (%) of total identified compounds

				Irradiation dose (kGy)				
No.	RT (min)	Compounds name	Classification	0	5	10	15	20
1	4.41	α-Pinene	Monoterpene hydrocarbon	0.12	0.17	0.19	0.21	0.24
2	6.13	α-Myrcene	Monoterpene hydrocarbon	7.08	7.58	7.88	7.94	8.05
3	7.35	o-Cymene	Monoterpene hydrocarbon	0.74	0.78	0.78	0.84	0.88
4	7.43	D-Limonene	Monoterpene hydrocarbon	0.50	0.55	0.59	0.60	0.62
5	8.00	cis-Ocemene	Monoterpene hydrocarbon	2.53	1.83	1.33	1.23	0.90
6	9.51	trans-Linalool Oxide	Oxygenated monoterpene	0.40	0.41	0.42	0.49	0.51
7	10.04	3,7-Dimethyl-1-ocyanol	Oxygenated monoterpene	0.65	0.66	0.69	0.70	0.75
8	10.36	trans-3-Caren-2-ol	Oxygenated monoterpene	0.49	0.50	0.52	0.54	0.59
9	12.08	2,2-Dimethylocta-3,4-dienal	Oxygenated monoterpene	0.46	0.48	0.50	0.50	0.53
10	12.20	Citronella	Oxygenated monoterpene	0.60	0.68	0.70	0.73	0.75
11	13.38	cis-Verbenol	Oxygenated monoterpene	3.15	2.78	1.62	1.01	1.04
12	14.04	Bornel	Oxygenated monoterpene	1.60	1.65	1.70	1.70	1.76
13	15.17	Nerol	Oxygenated monoterpene	0.60	0.68	0.70	0.73	0.75
14	15.29	Citronellol	Oxygenated monoterpene	0.80	0.89	0.96	1.01	1.01
15	15.90	Citral B (Neral)	Oxygenated monoterpene	31.85	32.73	32.89	33.00	33.03
16	16.28	Geraniol	Oxygenated monoterpene	4.40	4.47	4.88	5.00	5.02
17	17.21	Citral A (Geranial)	Oxygenated monoterpene	38.72	39.56	39.86	40.06	40.06
18	21.70	Geranyl acetate	Oxygenated monoterpene	0.79	0.89	0.95	1.00	1.03
19	23.00	trans-Caryophellene	Sesquiterpene hydrocarbon	0.65	0.66	0.67	0.70	0.70
20	29.62	Caryophyllene oxide	Oxygenated sesquiterpene	0.75	0.77	0.79	0.82	0.87
21	31.31	Juniper Camphor	Oxygenated sesquiterpene	0.44	0.49	0.51	0.53	0.60
22	47.15	Phytol	Diterpene alcohol	0.22	0.29	0.30	0.30	0.31

Total

Monoterpene hydrocarbons = 12 %

Oxygenated monoterpenes = 85 %

Sesquiterpenes + Diterpene = 3 %

In contrast, some components showed a notable decrease in response to gamma irradiation. Cis-ocemene, a monoterpene hydrocarbon, exhibited a consistent decline from 2.53% at 0 kGy to 0.90% at 20 kGy, indicating that degradation or transformation under oxidative conditions was induced by ionizing radiation. Cis-Verbenol, an oxygenated monoterpenoid, significantly decreased from 3.15% to 1.04% across the same irradiation range, with the most pronounced drop observed between 5 and 10 kGy (2.78% to 1.62%).

Several minor constituents demonstrated modest yet consistent increases across irradiation doses. Geraniol (4.40% to 5.02%) is an oxygenated monoterpene known for its antimicrobial and antioxidant properties. Citronellol (0.80% to 1.01%) and nerol (0.60% to 0.75%) are key fragrances and functional compounds. Caryophyllene oxide (0.75% to 0.87%) and trans-caryophyllene (0.65% to 0.70%) are both sesquiterpene derivatives. Other compounds, such as α-pinene, o-cymene, D-limonene, trans-linalool oxide, trans-3-caren-2-ol, and phytol, also showed slight but consistent enhancements with increasing irradiation, indicating potential radiosensitization or increased extraction efficiency under irradiation stress. Interestingly, the concentrations of most compounds tended to plateau between 15 and 20 kGy, suggesting that further increases in irradiation dose beyond 15 kGy may not result in proportional increases or decreases in compound abundance. This trend was especially evident in citral A, citral B, and bornel, indicating a potential threshold or saturation point for radiation-induced chemical modulation.

In conclusion, the application of gamma irradiation significantly altered the volatile profile of CCEO. Notably, irradiation at moderate to high doses (10–20 kGy) enhanced the concentrations of the most valuable aroma and bioactive compounds, citral A, citral B, and α-myrcene, while reducing the levels of radiation-sensitive constituents like cis-ocemene and cis-verbenol. These shifts highlight gamma irradiation as an encouraging tool for optimizing the chemical quality and potential therapeutic efficacy of CCEO.

### Total phenolic content (TPC) and total flavonoid content (TFC) of CCEO

**Fig. 2:**
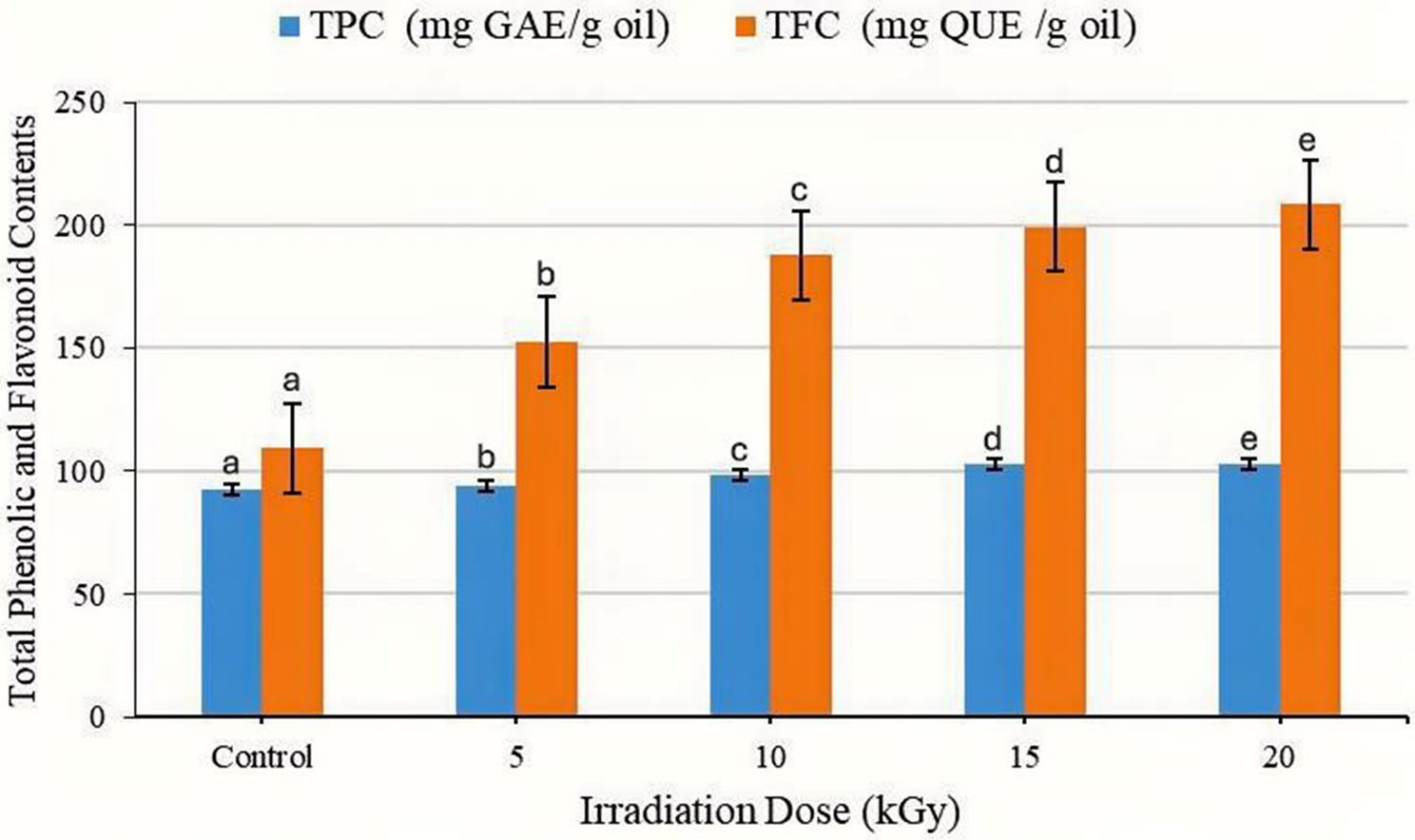
Effects of γ-irradiation at various doses (control, 5, 10, 15 and 20 kGy) on the total phenolic content (mg GAE/g oil) and total flavonoid content (mg QUE/g oil) of non-irradiated and irradiated CCEOs. Data are presented as the means ± standard errors (*n* = 3) at *p* < 0.05. Bars represent standard errors. Different letters indicate significant differences. γ-irradiation unit Gray (Gy)

The effects of GI on the TPC and TFC of CCEO were determined for various doses of radiation (0–20 kGy). The results, as shown in Fig. 2 indicated an increase in TPC and TFC with increasing radiation dose. The TPC values increased from 92.12 mg GAE/g oil in the control sample to 103.11 mg GAE/g oil at the maximum radiation dose (20 kGy), representing an overall rise of approximately 11.9%. A similar way was observed for the TFC, which significantly increased from 109.25 mg QUE/g oil in the control to 208.47 mg QUE/g oil at 20 kGy, reflecting a 90.7% increase.

### Antioxidant activity of CCEO

**Fig. 3:**
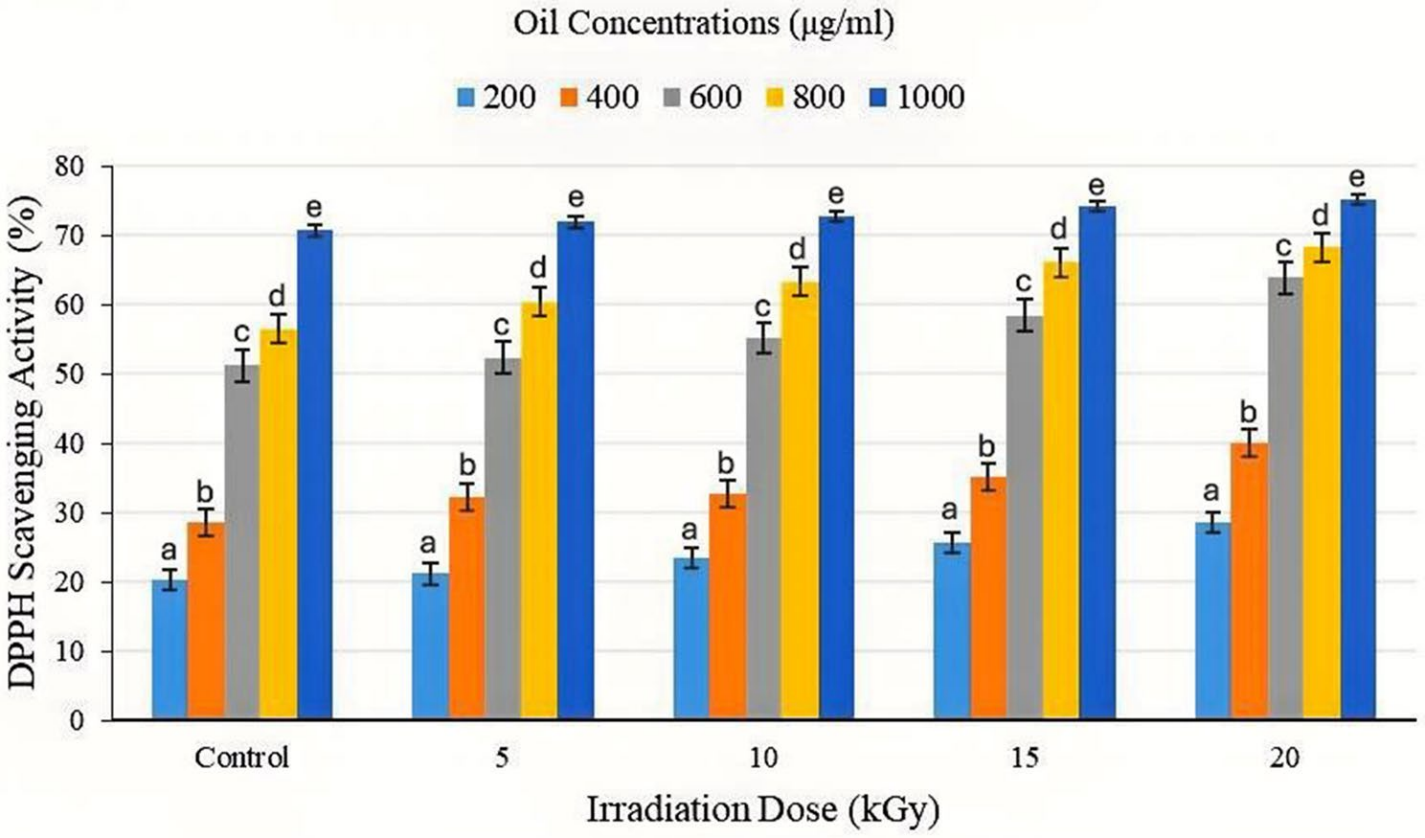
Effects of γ-irradiation at different doses (5, 10, 15 and 20 kGy) on the DPPH scavenging activity (%) of different CCEO concentrations (200, 400, 600, 800 and 1000 µg/mL). Data are presented as the means ± standard errors (*n* = 3) at *p* < 0.05. Bars represent standard errors. Different letters indicate significant differences

**Fig. 4:**
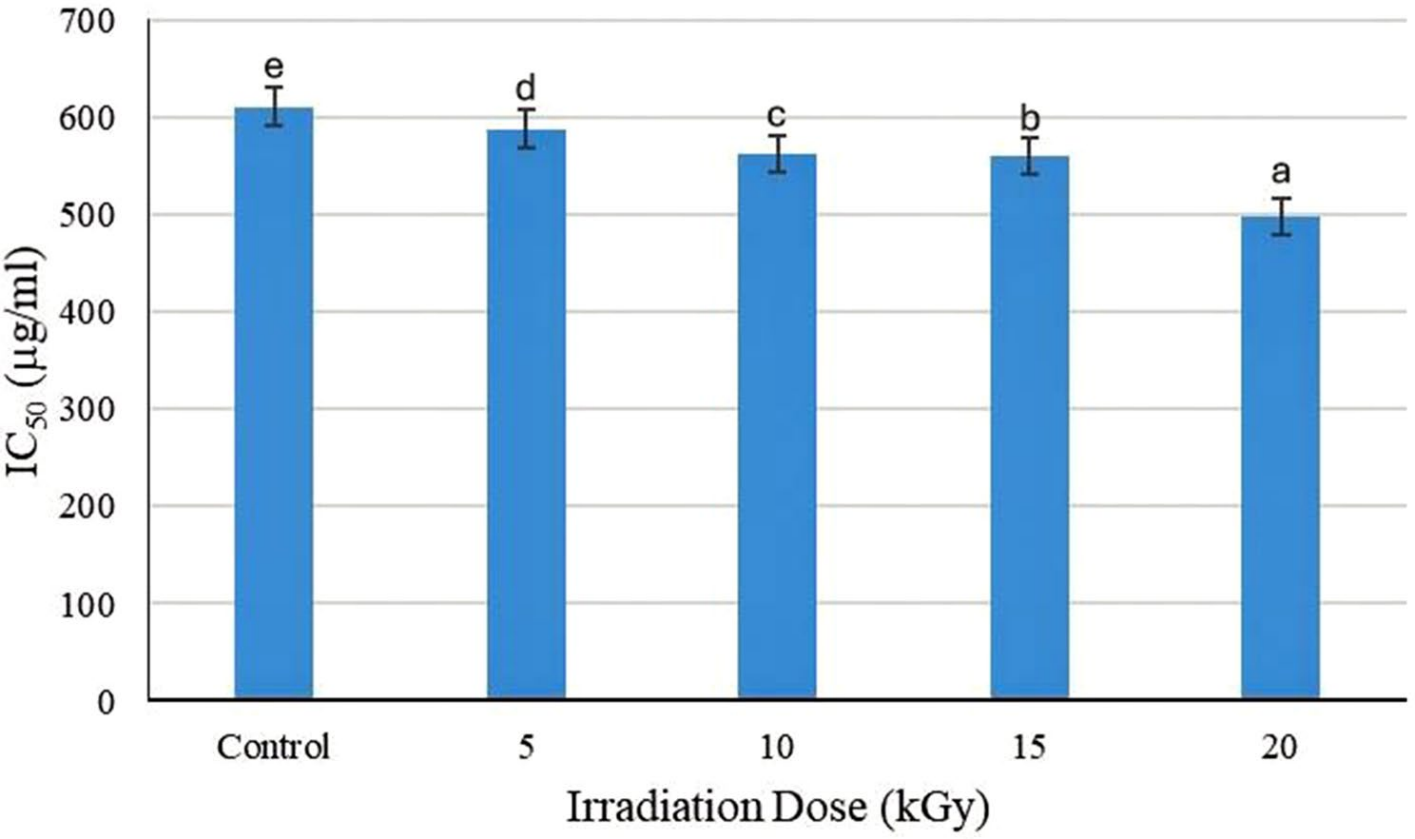
Effect of γ-irradiation at various doses (5, 10, 15 and 20 kGy) on the radical scavenging activity IC_50_ (µg/mL) of CCEO. Data are presented as the means ± standard errors (*n* = 3) at *p* < 0.05. Bars represent standard errors. Different letters indicate significant differences

Gamma-impact irradiation’s on the antioxidant activity of CCEO were evaluated at different irradiation doses (0, 5, 10, 15 and 20 kGy), and the results are summarized in Figs. 3 and 4, which show the free radical scavenging capacity of CCEO according to the IC_50_ values (the concentration of essential oil needed to restrain 50% of the radical efficacy).

The control sample (0 kGy) exhibited an IC_50_ value of 611.28 µg/mL, indicating that the highest concentration needed to achieve 50% inhibition. This value decreased progressively with increasing radiation dose, reaching 498.08 µg/mL at 20 kGy. This reduction in the IC_50_ suggests a development in the antioxidant potential of CCEO upon irradiation. Gamma irradiation resulted in a noteworthy rise in the antioxidant activity of CCEO at different doses. The highest doses at 1000 µg/mL were 71.89, 72.66, 74.20 and 75.15 µg/mL for 5, 10, 15 and 20 kGy, respectively, compared with the control at 70.66 µg/mL. At 400 µg/mL, the antioxidant activity slightly increased as a result of the irradiation dose of CCEOs (32.18, 32.68, 35.18 and 40.08 µg/mL) at 5, 10, 15 and 20 kGy, respectively, compared with the control (28.57 µg/mL). The IC_50_ content is declined significantly under γ-irradiation by 588.36, 562.14, 560.37, and 498.08 µg/mL oil for 5, 10, 15 and 20 kGy, respectively, compared with the control value of 611.28 µg/mL oil.

### Antibacterial activity of CCEO

The antibacterial activity of non-irradiated (0 kGy) and irradiated (5, 10, 15 and 20 kGy) CCEO was evaluated against two microbial strains, *P. aeruginosa* and *S. aureus*, and the outcomes are illustrated in Table 2. The results displayed as inhibition zone diameters (mm) indicating that gamma irradiation enhances the antimicrobial effectiveness of the CCEO, particularly against *P. aeruginosa*.

**Table 2:** Effects of γ-irradiation at different doses (5, 10, 15 and 20 kGy) on the antibacterial activity [inhibition zone diameter (mm)] of CCEO

Gamma Dose (kGy)	Inhibition zone (mm)							
	Microbial strain				Standard			
					GN		NS	
	*P. aeruginosa*	% Increase vs. Control	*S. aureus*	% Increase vs. Control	*P. aeruginosa*	*S. aureus*	*P. aeruginosa*	*S. aureus*
0 (Control)	8.1 ± 0.42^a^	—	10.2 ± 0.5^a^	—	2.21 ± 0.33^a^	2.54 ± 0.21^a^	2.52 ± 0.22^a^	1.85 ± 0.12^a^
5	11.3 ± 0.51^b^	+ 40%	14.5 ± 0.61^b^	+ 42%	2.88 ± 0.41^b^	3.23 ± 0.25^b^	NT	2.02 ± 0.14^b^
10	14.7 ± 0.62^c^	+ 81%	17.8 ± 0.76^c^	+ 75%	3.21 ± 0.38^c^	3.88 ± 0.19^c^	2.77 ± 0.24^c^	2.35 ± 0.15^c^
15	16.8 ± 0.55^d^	+ 107%	19.6 ± 0.60^d^	+ 92%	3.98 ± 0.48^d^	6.25 ± 0.23^d^	3.04 ± 0.27^d^	2.98 ± 0.14^d^
20	18.9 ± 0.74^e^	+ 134%	21.4 ± 0.82^e^	+ 110%	4.21 ± 0.62^e^	6.56 ± 0.31^e^	3.33 ± 0.29^e^	NT

Values are the means ± SD (n= 3). The percentage increase was calculated relative to the control (0 kGy) for the same bacterial strain. Different letters indicate significant differences (*p* < 0.05). Gentamycin (GN 10 μg), Nystatin (NS 100 μg), NT: not tested

The antibacterial activity, expressed as the inhibition zone diameter (mm), significantly increased in a way that is dose-dependent following gamma irradiation. Both *P. aeruginosa* and *S. aureus* demonstrated low inhibition zones in the control (non-irradiated) samples, with mean values of 8.1 mm and 10.2 mm, respectively. At 5 kGy, there was a noticeable increase in the inhibition zones: 11.3 mm for *P. aeruginosa* (+ 40% vs. control) and 14.5 mm for *S. aureus* (+ 42%). These increases suggest that even low gamma doses can enhance the antibacterial potency of the treated samples. The inhibitory activity continued to rise at 10 kGy, where *P. aeruginosa* and *S. aureus* exhibited inhibition zones of 14.7 mm (+ 81%) and 17.8 mm (+ 75%), respectively. A further increase was recorded at 15 kGy, with 16.8 mm for *P. aeruginosa* (+ 107%) and 19.6 mm for *S. aureus* (+ 92%).

The highest antibacterial activity was observed at 20 kGy, where the inhibition zones reached 18.9 mm (+ 134%) for *P. aeruginosa* and 21.4 mm (+ 110%) for *S. aureus*. The results clearly demonstrated that gamma irradiation intensified the antibacterial effectiveness of the sample in a way that is dose-dependent for both bacterial strains. Gamma irradiation between 5 and 20 kGy significantly enhanced antibacterial activity, with inhibition zones more than doubling at 20 kGy for *P. aeruginosa* and increasing by more than 100% for *S. aureus* compared to non-irradiated controls.

## Discussion

### Gamma irradiation and essential oil composition

Gamma (γ) irradiation causes radiolysis of intracellular water, leading to the formation of reactive oxygen species (ROS) such as superoxide anion (O_2_^•^), hydrogen peroxide (H_2_O_2_), and hydroxyl radicals (^•^OH). These ROS act not just as oxidative stressors but also as secondary messengers that modulate gene expression and activate plant defense mechanisms [7]. In aromatic and medicinal plants, the oxidative burst initiated by γ-irradiation triggers phenylpropanoid metabolism through the up-regulation of PAL, chalcone synthase (CHS), and terpene synthase (TPS) enzymes, thereby enhancing the biosynthesis of phenolic compounds, flavonoids, and oxygenated monoterpenes [6, 8].

Gamma irradiation significantly modified the chemical profile of CCEO, particularly by increasing oxygenated monoterpenes such as citral (neral + geranial) and geraniol at moderate doses (10–15 kGy). These effects are attributed to low-dose oxidative signaling that activates key enzymes in the terpene pathway, including monoterpene synthases and hydroxylases, leading to enhanced formation of geranial and related oxygenated derivatives [16]. At 20 kGy, the decline in citral and β-myrcene content likely results from excessive oxidation or volatilization, consistent with compositional losses reported in other irradiated aromatic herbs [26]. Studies using GC-MS and GC-IMS analyses confirm that irradiation primarily causes oxidation, dehydrogenation, and isomerization rather than generating novel compounds, leading to a higher citral: β-myrcene ratio and enhanced antioxidant potential at moderate doses [13, 27]. Comparable irradiation-induced enrichment of oxygenated terpenes has been reported for *Satureja mutica* and thyme, where 2.5–15 kGy promoted monoterpene biosynthesis and antioxidant potential before degradation occurred at higher exposures [11, 14].

### Total phenolic and flavonoid contents of CCEO

Total phenolic (TPC) and flavonoid (TFC) contents increased up to 20 kGy, revealing a typical hermetic pattern. The observed rise in TPC and TFC following γ-irradiation thus reflects a ROS driven activation of secondary metabolite pathways, improving antioxidant performance of the irradiated oil [6, 28]. Under stress conditions (γ-irradiation), plants may specifically upregulate the biosynthesis of certain flavonoid subclasses, such as flavonols, due to their crucial role in combating oxidative stress induced by radiation [29, 30]. The increase in TPC and TFC at 10, 15, and 20 kGy likely stems from the activation of PAL and chalcone synthase under mild oxidative stress, boosting secondary metabolite flux [11]. Such γ-dose-dependent modulation of antioxidant metabolites has been widely interpreted as a controlled stress-signaling phenomenon rather than a destructive effect [31]. Similar responses have been described in *Satureja mutica* and thyme, where moderate doses enhanced phenolic accumulation while excessive irradiation impaired biosynthetic enzymes [14]. Comparable findings were observed in *Ferula gummosa*, where PAL activation under 10–15 kGy irradiation enhanced phenolic synthesis [32]. Studies on *Thymus pallescens* also demonstrated that 5–10 kGy exposure modified monoterpene and sesquiterpene ratios, correlating with stronger antioxidant and insecticidal properties [33]. Low to moderate doses appear to trigger defense-related enzyme systems without causing oxidative collapse. In *Thymus vulgaris* and *Mentha spicata*, TPC and TFC rose up to 15 kGy and declined thereafter [34]. These reports support the current results indicating that 10–20 kGy lies within the stimulatory, not deleterious, range for *C. citratus*. Conversely, at higher doses, ROS overproduction may trigger phenolic degradation or polymerization, reducing extractable content. Ammendola et al., (2020) [35] found that gamma radiation led to a loss of TPC and TFC in *Cistus* extracts, suggesting potential degradation of high molecular weight compounds and a decrease in antioxidant capacity due to free radical formation. Similarly, *Phaseolus vulgaris* leaves showed a decrease in preformed polyphenolic flavonoids due to gamma radiation-induced oxidative stress [36]. Furthermore, other non-flavonoid phenolic compounds might respond differently to gamma radiation. Some may remain stable, decrease, or even be degraded. Pereira et al., (2018) [37] showed that heterogeneous effects of gamma radiation on phenolic profiles, with increases in some compounds and decreases in others. While certain flavonoids might increase, other preformed polyphenolic flavonoids could decrease, or other classes like hydroxycinnamates might show compensatory responses, indicating a dynamic metabolic shift rather than a uniform increase across all phenolics [36].

### Antioxidant activity and radical scavenging efficiency of CCEO

The observed reduction in IC_50_ values and increased DPPH scavenging efficiency at 5–20 kGy correlate with both the rise in TPC/TFC and the enrichment of oxygenated monoterpenes. Oxygenated terpenes such as citral, geraniol, and β-myrcene exhibit strong hydrogen donating capacity through conjugated C = C and hydroxyl groups. Moderate γ-irradiation likely converted non-oxygenated monoterpenes into more reactive aldehydes and alcohols, increasing total radical scavenging efficiency [38]. Hadjadj and Hazzit (2020) [39] stated that since hydroxyl and phenolic groups are known to be crucial for antioxidant activity, the increase in inactive compounds in EOs that contain monoterpene hydrocarbons with a phenolic ring and oxygenated monoterpenes, which contain both of these groups, may be the cause of DPPH scavenging activity in an irradiation dose-dependent manner. Additionally, *P*-cymene exhibits substantial antioxidant effects when it interacts with other monoterpenes, creating synergistic interactions that help elucidate the potent antioxidant impact of EOs [40]. The synergy between phenolic antioxidants and oxidized terpenes likely accounts for the marked improvement in free radical quenching ability. Comparable results have been reported for irradiated celery seed and thyme essential oils, where 5–15 kGy treatments significantly increased antioxidant indices [11, 26]. Similar findings were reported in *Curcuma longa* and *Curcuma alismatifolia*, where moderate irradiation (≤ 15 Gy) enhanced phenolic and flavonoid accumulation, resulting in improved DPPH radical scavenging activity [13, 41]. A study by Do et al., (2021) [42] involving a DPPH free radical scavenging experiment revealed that the antioxidant capacity of CCEO and their fractions varied in terms of percentage inhibition following distillation, with the fraction having a greater phenolic content indicating a greater antioxidant capacity. These findings reinforce that moderate γ-doses optimize oxidative stress responses, maximizing redox functionality without molecular degradation.

### Antibacterial activity of CCEO

A clear dose-dependent enhancement in antibacterial efficacy was observed, at 20 kGy treatment producing the largest inhibition zones against *Staphylococcus aureus* and *Pseudomonas aeruginosa*. The increased activity of irradiated CCEO is primarily linked to elevated citral and geraniol levels, which disrupt bacterial membranes, increase permeability, alter proton-motive force, interfere with oxidative phosphorylation, and inhibit key enzymes of energy metabolism [43, 44]. Gram-positive bacteria were more susceptible than Gram-negative strains, likely because the lipophilic monoterpenes penetrate the thicker peptidoglycan matrix more easily than the outer lipopolysaccharide barrier of Gram-negative cells [45]. The CCEO contain a large number of lipophilic substances that may thaw in the biomembranes of microorganisms and interact with lipids and proteins to cause cell disturbance, infiltration of cell contents, and cell death [46].

The comparative analysis reveals that the biological properties are not merely inherent to the species but are profoundly influenced by its specific chemical profile or chemotype. For example, while *Cymbopogon giganteus* essential oil demonstrated antimicrobial effects against a range of microorganisms, *Cymbopogon citratus* essential oil failed to inhibit *Pseudomonas aeruginosa* highlighting species-specific efficacy linked to compositional differences [47]. Similarly, study on *Cymbopogon flexuosus* essential oil and pure citral against *Acinetobacter baumannii* indicated that citral itself possessed lower minimum inhibitory and bactericidal concentrations than the whole essential oil, emphasizing citral’s significant contribution to the antimicrobial effect [48]. Similar γ-induced antimicrobial improvements have been documented in celery seed and *Satureja mutica* essential oils [14, 26].

However, the antibacterial efficacy is not always attributable to a single compound. For instance, studies on *Cymbopogon khasianus* essential oil suggest that while geraniol is a major constituent, it is not solely responsible for antimicrobial activity; other components like geranyl acetate, linalool, and β-ocemene also play a role [49]. This suggests complex synergistic or additive interactions among the various constituents. Furthermore, a comparison of *C. citratus* and *C. proximus* essential oils showed that CCEO, rich in geranial and neral, exhibited significantly higher antifungal and antibiofilm activities compared to *C. proximus* EO, whose major compounds were piperitone and α-terpinolene [50].

Elmi et al., (2025) [51] explored the antibacterial potency and phytochemical insights of various *Cymbopogon* essential oils, including *C. citratus*, highlighting their potential as natural antimicrobial agents. Geraniol has been shown to possess strong antimicrobial properties, effectively inhibiting bacterial growth and exhibiting cytotoxic effects against cancer cells [52]. Citral has been shown to modulate virulence factors in methicillin-resistant *Staphylococcus aureus* [53]. Citrus peel essential oils exposed to 0–10 kGy showed significant increases in antioxidant capacity and maximal inhibition zones at 4 kGy, particularly for lemon oil, again illustrating that moderate irradiation can potentiate antimicrobial function [54].

Our study showed that gamma irradiation could effectively modulate the concentrations of geranial, neral, α-myrcene, and geraniol in CCEO providing a powerful method for influencing its chemotype. This controlled alteration of the phytochemical profile offers a strategic approach to enhancing desired pharmacological properties, such as the observed improvements in antioxidant capacity and heightened antibacterial efficacy against *P. aeruginosa and S. aureus*. Therefore, understanding and actively modulating the chemical composition through techniques like gamma irradiation positions it as a valuable tool for tailoring the functional properties and optimizing the efficacy of natural products.

## Conclusion

This study demonstrated that controlled γ-irradiation could modulate the phytochemical and functional characteristics of *Cymbopogon citratus* essential oil (CCEO) without compromising its integrity. Irradiation at 5, 10, 15, and 20 kGy induced measurable alterations in essential oil composition, particularly enhancing oxygenated monoterpenes such as citral (neral + geranial), β-myrcene, and geraniol, which are largely responsible for CCEO’s biological activity.

Concurrently, a dose-dependent rise was noted in the TPC and TFC, which corresponded with improved antioxidant capacity, as reflected by lower IC_50_ values and stronger DPPH scavenging activity. Furthermore, the antibacterial assays confirmed that irradiated CCEO exhibited heightened inhibitory effects against both *S. aureus* and *P. aeruginosa*, with the highest efficacy recorded at 20 kGy. This indicates that qualitative modification of monoterpene structures underlies the observed enhancement in bioactivity. The optimal γ-irradiation range (10–20 kGy) therefore represents a balance between biochemical activation and the prevention of oxidative deterioration.

Collectively, these findings highlight γ-irradiation as a green, residue-free, and reproducible postharvest technology capable of enhancing the value and efficacy of medicinal and aromatic plant products. The γ-irradiated lemongrass essential oil shows promise as a natural antioxidant and antimicrobial agent for use in food preservation, cosmetic formulations, and pharmaceutical products.

## Data Availability

No datasets were generated or analysed during the current study.
